# An Affordable Streamflow Measurement Technique Based on Delay and Sum Beamforming

**DOI:** 10.3390/s22082843

**Published:** 2022-04-07

**Authors:** Giuseppe Passarella, Aimé Lay-Ekuakille, John Peter Djungha Okitadiowo, Rita Masciale, Silvia Brigida, Raffaella Matarrese, Ivan Portoghese, Tommaso Isernia, Luciano Blois

**Affiliations:** 1 Water Research Institute, National Research Council (IRSA-CNR), 70132 Bari, Italy; giuseppe.passarella@ba.irsa.cnr.it (G.P.); silvia.brigida@ba.irsa.cnr.it (S.B.); raffaella.matarrese@ba.irsa.cnr.it (R.M.); ivan.portoghese@ba.irsa.cnr.it (I.P.); 2 Department of Innovation Engineering, University of Salento, 73100 Lecce, Italy; aime.lay.ekuakille@unisalento.it; 3 Department of Information Engineering, Infrastructure and Sustainable Energy (DIIES), University “Mediterranean” of Reggio Calabria, 89124 Reggio Calabria, Italy; johnpdjungha@unirc.it (J.P.D.O.); tommaso.isernia@unirc.it (T.I.); 4 Department of Engineering Sciences, University of Rome “Guglielmo Marconi”, 00193 Rome, Italy; l.blois@unimarconi.it

**Keywords:** sensing systems for hydrodynamics, beamforming for imaging, doppler processing, MUSIC technique, synthetic aperture radar, channel flow characterization, flow measurement

## Abstract

At the local scale, environmental parameters often require monitoring by means of affordable measuring techniques and technologies given they need to be frequently surveyed. Streamflow in riverbeds or in channels is a hydrological variable that needs to be monitored in order to keep the runoff regimes under control and somehow forecast floods, allowing prevention of damage for people and infrastructure. Moreover, measuring such a variable is always extremely important for the knowledge of the environmental status of connected aquatic ecosystems. This paper presents a new approach to assessing hydrodynamic features related to a given channel by means of a beamforming technique that was applied to video sensing. Different features have been estimated, namely the flow velocity, the temperature, and the riverbed movements. The applied beamforming technique works on a modified sum and delay method, also using the Multiple Signal Classification algorithm (MUSIC), by acting as Synthetic Aperture Radar (SAR) post-processing. The results are very interesting, especially compared to the on-site measured data and encourage the use of affordable video sensors located along the channel or river course for monitoring purposes. The paper also illustrates the use of beamforming measurements to be calibrated by means of conventional techniques with more accurate data. Certainly, the results can be improved; however, they indicate some margins of improvements and updates. As metrics of assessment, a histogram of greyscale/pixels was adopted, taking into account the example of layers and curve plots. They show changes according to the locations where the supporting videos were obtained.

## 1. Introduction

The European Union’s Water Framework Directive (WFD) explicitly acknowledges the importance of the flow regime for the quantitative and qualitative evolution dynamics of natural water systems.

The analysis of relevant flow components and their alteration can, therefore, be used to derive reliable indicators of hydrological impact on the aquatic systems and ecology [1] to be used for the implementation of the River Basin Management Plan (RBMP).

Nevertheless, the application of such analysis often implies the use of large existing data sets and long time series to be processed by statistical or physically based models. This data need represents a weakness point, particularly for smaller watercourses, where streamflow measurements are often scarce in space and time or even missing [2]. Monitoring programs should be adapted to provide an improved picture of hydrological alterations and their impact on qualitative and morphological watercourse features including smaller ones (small parts of creeks, headwaters, little rivers, channels).

As a proof of the need to improve the natural water body monitoring systems, the International Association of Hydrological Sciences (IAHS) declared the period 2003–2012 as the “decade of the ungauged basin” [3], promoting the development of science and technology to provide hydrological data where the ground-based observations are needed but missing.

Establishing an effective hydrological monitoring network often requires conciliation between the ideal solution and the requirement for cost-effectiveness [4]. In the Mediterranean environment, monitoring requirements, and thus costs, are usually higher compared to central and northern European basins, due to high spatial and temporal variability of the hydrological regime in such areas as a result of hydrogeological and climatic factors.

One key issue in arid land hydrology is the characterization of riverbed infiltration under different hydrologic, hydraulic and sediment-load regimes. In such environments, the interaction between the streamflow and the vadose zone must be thoroughly investigated for the appropriate design and development of flow measurement networks.

In arid and semi-arid regions, aquifers are often the principal water supply and it is frequently asserted that infiltration through streambeds during flood events is the main form of recharge [5,6,7]. Review papers exist for groundwater recharge in general [8,9], but not specifically for the ephemeral and intermittent streams characteristic of arid systems. Shanafield and Cook [10] report a simple method to provide estimates of streambed infiltration to groundwater based on differential discharge measurements between upstream and downstream sections. Nevertheless, in ephemeral watercourses where flow is rarely stable, loss rates need to be determined by integrating the upstream and downstream flow rate over the entire flow event [11].

Flow is generally measured automatically using rating curves to relate flow depth to discharge rate but determining accurate rating curves in ephemeral streams is challenging. In ephemeral and intermittent stream basins, water quickly runs into streams, leading to significant erosion and deposition phenomena producing, in turn, changes in the streambed geometry and errors in flowrate measures [12]. An innovative and reliable alternative to the traditional riverbed measurement methods consists of using sensing systems capable of monitoring flow conditions and other possible geo-environmental parameters based on still or video imaging [13,14].

In this paper, a complex case study is proposed related to the Canale Reale, an ephemeral watercourse located in a typical Mediterranean environment of southeastern Italy and characterized by strong anthropogenic pressures and a lack of hydrological data. A significant anthropogenic component of the considered case study consists of the allowed practice of discharging treated wastewater from some nearby water treatment plants into the stream. Although this practice may lead to the assumption that it produces ecological and quality impairments to the receiving river, several studies showed that effluent could be an important source of environmental base flow for dried-out streams because of over-withdrawals and climate change, particularly in semi-arid and arid areas [15].

In this general context, we propose an affordable and reliable measurement technique, based on beamforming [16,17,18,19,20] applied on video sensing, to estimate some hydrodynamic features of the considered watercourse.

The digital beamforming synthetic aperture radar approach is one of the ways to form a beam that collects information from an array of sensors. In general, beamforming is applied in many fields close to the interest of this paper [21,22,23,24]. The concept of array processing is the base of beamforming that encompasses the following main issues: array configuration, temporal and spatial characteristics of the signal and interference, and finally the objective of the array processing. The radar systems generally work using antenna arrays, and they are the first application of beamforming; the antenna arrays are also used to find the direction of beams/signals, such as the direction of arrival (DOA) [25,26].

The ultimate aim of the proposed technique consists of creating an effective monitoring system capable of carrying out measurements spread along the channel, repeatable for different seasonal flow regimes.

## 2. Materials and Methods

### 2.1. Beamforming Imaging Based Algorithms

#### 2.1.1. MUSIC

**Figure 1:**
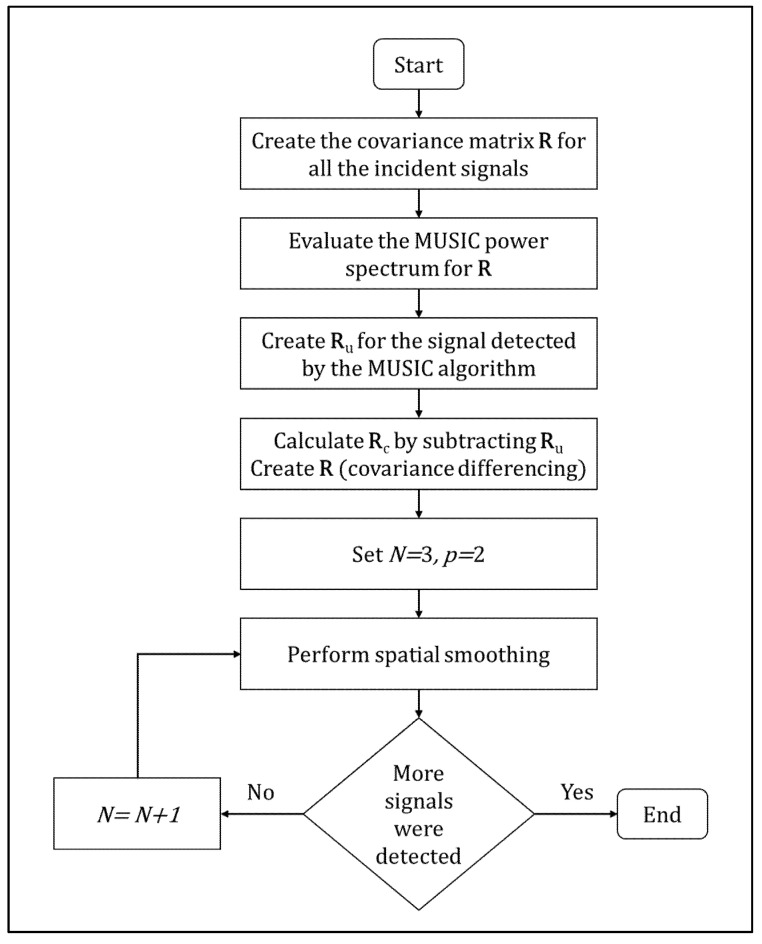
MUSIC algorithm carried out in the paper for detecting and separating peaks for close signals.

MUSIC, or multiple signal classification [27,28], is an algorithm used for frequency estimation and transmitter location. This algorithm makes it possible to determine the direction of signals present on a sensor network even when the signal-to-noise ratio is very low. Figure 1 shows the MUSIC algorithm used in this paper for detecting and separating peaks for close signals.

We assume that a signal vector consists of complex exponentials, whose frequencies are unknown, in the presence of white Gaussian noise as given by the linear model

(1) X=As+n

Here, $A=\left[a\left(\omega_{1}\right),\ldots ,a\left(\omega_{p}\right)\right]$ is an M×p Vandermonde matrix of steering vectors $a\left(\omega \right)={\left[+1,e^{-j\omega }e^{-j2\omega },\ldots ,e^{-j\left(M-1\right)\omega }\right]}^{T}$ and $S=\left[s_{1},\ldots ,\;s_{p}\right]$ is the amplitude vector. A crucial assumption is that the number of sources P, is less than the number of elements in the measurement vector, M, i.e., P<M.

The M×M autocorrelation matrix of X is then given by $R_{x}=AR_{s}R^{H}+\sigma^{2}I$.

Where, $\sigma^{2}$ is the noise variance, I is the M×M identity matrix, and $R_{s}$ is the p×p autocorrelation matrix of S.

(2) $${\hat{R}}_{x}=\frac{1}{N}XX^{H},$$

where N>N is the number of vector observations and $X=\left[X_{1},X_{2},\ldots ,X_{3}\right]$.

Given the estimation of $R_{x}$, MUSIC estimates the frequency content of the signal or autocorrelation matrix using an eigenspace method.

Since $R_{x}$ is a Hermitian matrix, all its M eigenvectors $\left\{V_{1},V_{2},\ldots ,V_{M}\right\}$ are orthogonal to each other. If the eigenvalues of $R_{x}$ are sorted in decreasing order, the eigenvectors $\left\{V_{1},V_{2},\ldots ,V_{p}\right\}$ corresponding to the p largest eigenvalues (i.e., directions of largest variability) span the signal subspace $\mu_{s}⊥\mu_{N}$.

##### Estimating the Exact Number of Targets

For the MUSIC algorithm to produce an accurate estimate of the DOA [29,30], the total number of targets in each range bin must be known.

##### Minimum Description Length

One algorithm that estimates the number of targets is called the minimum description length (MDL). MDL uses the eigenvalues of the correlation matrix and maximizes the following log likelihood ratio [31]

(3) $$L\Theta =-N\log\det R-tr{\left[R\right]}^{-1}\hat{R},$$

where $\hat{R}$ is the sample covariance matrix

(4) $$\hat{R}=\frac{1}{N}\sum_{i=1}^{N}\left(t_{i}\right)\times {\left(t_{i}\right)}^{H},$$

By substituting in maximum likelihood estimates, the log likelihood ratio is reduced to

(5) $$L\left(k\right)=\left(p-k\right)N\log\left(\frac{\prod_{i=k+1}^{P}l_{i}^{1/\left(p-k\right)}}{\frac{1}{p-k}\sum_{i=k+1}^{p}l_{i}}\right),$$

where p is the number of array elements, k is the number of targets, and $l_{i}$ are the eigenvalues with $l_{i}\geq l_{2},\ldots ,l_{p}$. Using this maximum likelihood estimate and adding in the free parameter calculation, the resulting criterion is

(6) $$MDK\left(k\right)=L\left(k\right)+\frac{1}{2}k\left(2p-k\right)\log N,$$

where N is the number of observations of the signal for our radar, the correlation matrix is composed ot the average of 6 pulses, so N=6. To solve the maximum likelihood estimation, L should be maximized. The number of targets is the value of k, minimizing the MDK. Once this has been done, the p−k eigenvectors corresponding to the p−k smallest eigenvalues can be used to from a noise matrix as explained above.

##### Eigenvalue Gradients

The gradients of the eigenvalues are used to estimate the number of targets. Given the eigenvalue decomposition of the correlation matrix, where L is the number of antenna elements, this results in eigenvalues [20,32]

(7) $$\lambda_{1}\geq \lambda_{2}\geq \cdots \geq \lambda_{p}\geq \cdots \geq \lambda_{L},$$

(8) $$\Delta \lambda =\lambda_{1}-\lambda_{L}/\left(L-1\right),$$

(9) $$\Delta =\lambda_{1}-\lambda_{i+1},\;\;for\;i=1,\ldots ,L-1,\;\;$$

Next it is found i that satisfies

(10) $$\Delta \lambda_{i}\leq \Delta \lambda ,\;\;$$

Then, i is taken as the first one of the last continuous block and the estimated signal number is

(11) $$p=i_{0}-1.$$

##### Detecting Moving Targets and Measuring Velocity

One of the most fundamental tasks of a radar is to be able to detect targets accurately and to measure the velocity if the target is moving. To do this accurately, we used the Array Digital Beamforming Algorithms technique with the specification called Doppler Processing [33,34].

Several simultaneous criteria are required for a signal to be considered a detection. It is an adaptive process that automatically adjusts to background noise and environmental influences. There is a test cell, where the surrounding cells are summed, multiplied by a constant and used to establish a threshold, as shown in Equation (12).

(12) $$Threshold\;criteria\left\{\begin{array}{c} \begin{array}{c} \{Cell\left(n\right)>[Cell\left(n-2\right)+\\ Cell\left(n-1\right)+\\ Cell\left(n+1\right)+ \end{array}\\ Cell\left(n+2\right)]\times \\ constant\} \end{array}\right\}\;\;$$

The detection only covers speeds that exceed the speed rejection setting. As an example of speed rejection, if speed rejection is set to 75 mph, hailstones travelling at 50 mph in a thunderstorm will not be detected, but an aircraft travelling at 100 mph will be, as illustrated in Equations (12) and (13).

(13) $$Peak\;criteria\left\{\begin{array}{c} \left(\frac{\Delta Amplitude}{\Delta Frequency}\right)Cell\left(n-1\right)<0\\ \left(\frac{\Delta Amplitude}{\Delta Frequency}\right)Cell\left(n-1\right)>0 \end{array}\right\}\;\;$$

(14) $$Speed\;criteria\left\{\left(\frac{C\times Doppler\;Frequency}{2\times Transmit\;Frequency}\right)\right\}>Rejection\;$$

This involves minimizing the probabilities of a false alarm (reporting a detection when there is no target) and missed detection (not reporting a detection when there is a target).

#### 2.1.2. Doppler Processing

**Figure 2:**
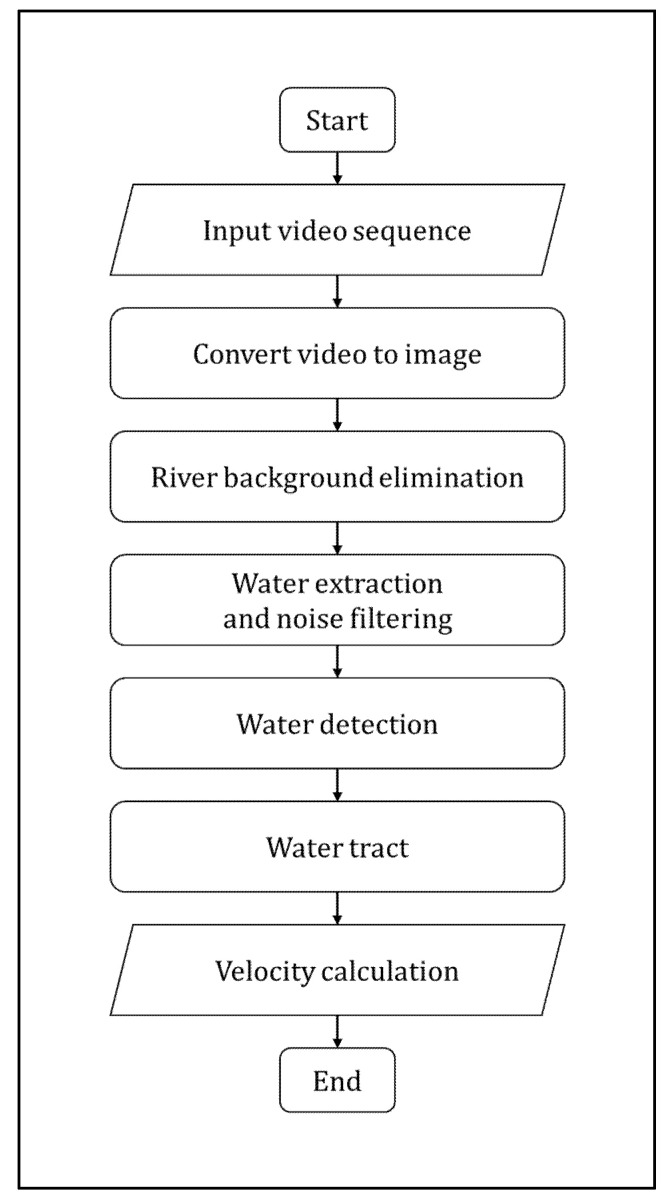
Doppler processing for characterizing physical features of the water channel.

Doppler processing is another important technique that uses Doppler information to detect targets and measure their speed [35]. This technique can detect targets when clutter is the dominant interference [34]. This is applicable in environments where echoes and clutter (buildings, trees, the movement of water waves, and other objects) can drown out the return signal. Increasing the transmit power does not help the detection of targets in clutter, as the power that returns from the clutter is also increased. Figure 2 shows the Doppler processing for characterizing physical features of the water channel

Doppler processing is the main technique for increasing the signal to clutter ratio (SCR). To increase the SCR, the signals must be frequency separable. The moving target signal can be separated from the clutter echoes in the frequency domain [36]. The clutter is relatively stationary (wind moving trees, flowing water, etc.) and, therefore, contributes little or nothing to the Doppler shift of the return echo. Thus, we can apply Doppler processing to detect significant clutter. Doppler processing has two general cases: moving target indication (MTI) and pulse Doppler processing. MTI requires fewer calculations but can only detect the presence of a moving target. MTI cannot identify if there is more than one target per range bin or what the speed of that target is. The main advantage of an MTI is less complexity and fewer calculations. On the other side, pulsed Doppler processing can detect a target similarly, but it can also measure the Doppler shift and, from this, determine the velocity of the target. A further advantage of pulsed Doppler processing is the detection of multiple targets, with the only condition that they are separated by a sufficiently large Doppler shift. These advantages bring with them the trade-off of increased computation and complexity. An MTI may only be implemented by making use of temporal data on many coherent, i.e., slow time, pulses, so the consecutive slow time intervals will be filtered out in the internal composition of digital beamforming using a high pass filter. This attenuates the clutter that is confined around 0 Hz. Finally, a threshold is applied to the time data and a decision is made on the detection.

## 3. Study Area

**Figure 3:**
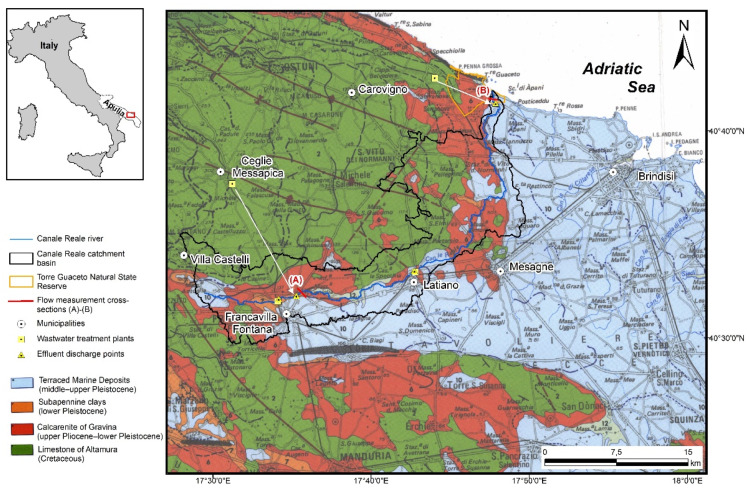
Study area and geological map (modified after Ciaranfi et al., 1988 [37]).

The proposed measurement technique for estimating hydrodynamic features of a watercourse was applied to the Canale Reale River, which is located in the south-eastern part of the Apulia region (Italy) (Figure 3) and classified as a heavily modified river with an ephemeral–intermittent flow regime. Due to the extension of its catchment area (approximately 210 km^2^), it represents the most significant watercourse in south Apulia. Along its course, almost 50 km long, it receives the treated effluents from wastewater treatment plants (WWTP) of four different municipalities: Ceglie Messapica, Francavilla Fontana, Latiano and Carovigno (Figure 3). The Canale Reale ends at the Natural State Reserve of Torre Guaceto where it partially feeds a coastal salty wetland.

### 3.1. Groundwater

The stratigraphic and structural setting of the area [37,38,39] (Figure 3) justifies the presence of two distinct aquifer structures: the deep aquifer hosted in the Cretaceous carbonate rock succession and the shallow porous aquifer, corresponding to sand-calcarenite levels of Terraced Marine deposits [40]. The latter, intensively used for local irrigation needs, is only recharged by rainwater and feeds the Canale Reale River and some other local topographically depressed areas.

The deep carbonate aquifer, affected by karst phenomena, is mainly recharged by rainfall that infiltrates the innermost part of the region. Generally, groundwater flows freely close to the coast where it gives rise to submarine and subaerial springs diffusely emerging in morphologically depressed zones. Particularly, along the Torre Guaceto wetland, mixing between the deep and shallow aquifers has been recognized [41]. The regional aquifers have been extensively monitored in space and time since the middle of the 1990s and various studies have been carried out on groundwater quality and quantity during the last decades [42].

### 3.2. Climate

**Figure 4:**
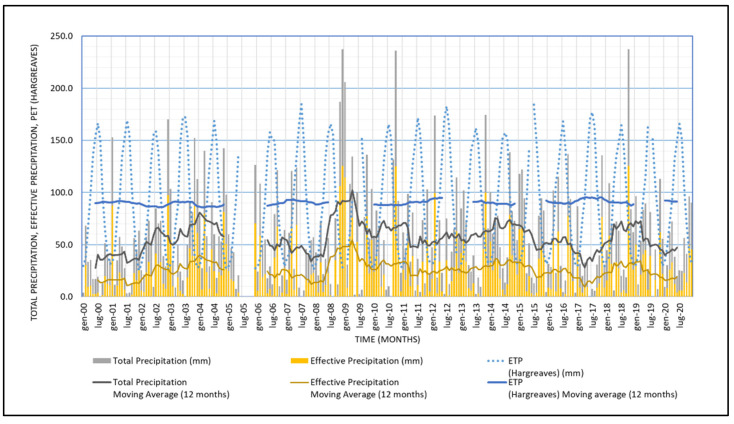
Monthly elements of the hydrological balance in the area under investigation. Effective precipitation and the potential evapotranspiration (PET) were assessed based on [50] and the Hargreaves formula [51], respectively. Precipitation and temperature data were downloaded from the Hydrological Annals published by the Apulian Civil Protection [52].

The study area is characterized by a typical dry sub-humid, Mediterranean climate, as for most of the region. More in detail, the precipitation is scarce, around 600 mm/year, on average, and temperatures are very mild overall, most of the year. Summers are long, dry, and hot; there can be a lack of rainfall for two or three consecutive months and temperature often exceeds 40 °C. In winter, precipitation is scarce and irregular, and temperatures are mild, usually exceeding 10 °C [43,44,45,46,47]. Figure 4 shows some elements of the hydrological balance in the area under investigation, during the last 20 years. In particular, the monthly measured total and estimated effective precipitation are plotted together with the estimated monthly potential evapotranspiration at the weather station of Latiano, located in the middle of the study area. During the considered period, the estimated PET was constantly around 1000 mm/year.

The non-parametric Mann–Kendall test for a monotonic trend, recommended by the World Meteorological Organization (WMO) [48,49], revealed no monotonic trends in any of the considered meteo-climatic parameters, during the considered period.

### 3.3. Main Environmental Issues

The flat morphology of the area favored intense agricultural exploitation, mainly for to olives, grapes, and vegetables. Intensive farming and the progressive diffusion of water-demanding crops over the last 50 years produced an increase in water uptake for irrigation purposes, with a significant anthropogenic impact on local groundwater resources, especially those hosted in the deep aquifer, both in quantitative and qualitative terms. Indeed, the huge withdrawals have caused an imbalance between fresh groundwater and the underlying salt water, with localized phenomena of saline contamination along the coastal and even in the inner areas [53]. This issue is also worsened by the effects of climatic changes that produce the succession of intense rainfall events and dry periods characterized by high evapotranspiration rates, which limit the natural aquifer recharge in turn [54]. Furthermore, the water balance is strongly addressed towards groundwater overexploitation given that surface watercourses are lacking or characterized by intermittent regimes.

### 3.4. In Field Measurement and Video

In order to apply and validate the proposed methodology, on-site flow video recording and measurements were carried out in three cross-sections along the Canale Reale River: one, named section A, and the other two, named B1 and B2, located less than 100 m downstream from the Ceglie Messapica and Carovigno WWTP effluent discharge points, respectively. Particularly, sections B1 and B2 are 8 m apart from each other.

**Figure 5:**
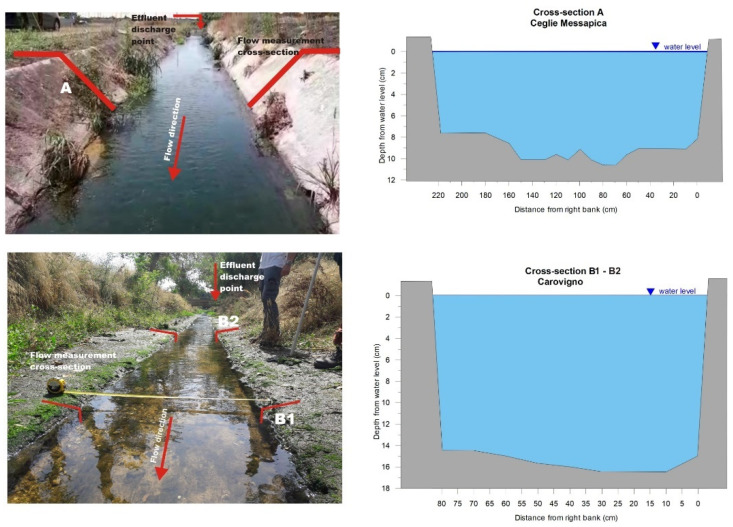
Flow measurement cross-sections of Ceglie Messapica (A) and Carovigno (B1 and B2) (for location see Figure 3). The surveyed geometry for cross-sections B1 and B2 is very similar, due to the regular shape of the banks in that river stretch.

**Figure 6:**
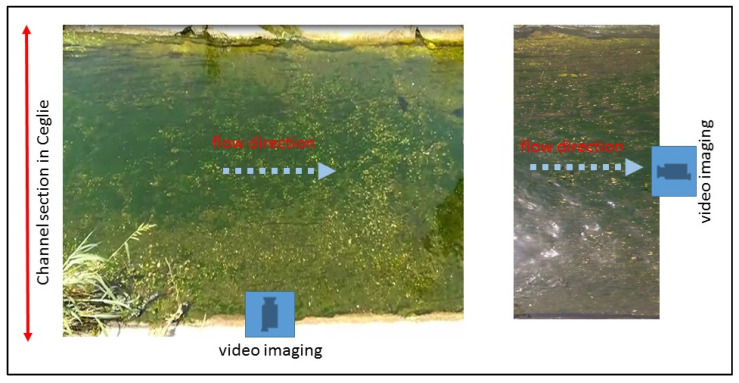
Example of channel photos used for processing (cross-section A).

Figure 5 shows the location and the surveyed geometry of the cross-sections, while Figure 6 shows, as an example, a still image of the water flow video through section A.

Concerning the on-site flow measurement methods, an exhaustive account of the related advantages and disadvantages is found in Dobriyal et al. [55]. In recent work, Portoghese et al. [56] compared two measurement methods, which are the more reliable current meters method (CM method) and the float method (F method). It resulted that the F method overestimates the water flow rate of the CM by about 11%. This relative error can be considered widely acceptable compared to the more rigorous, but much more time-consuming, CM method, especially when measurements need to be repeated in time. In this study, both methods were used in section A while only the CM method was used in sections B1 and B2. The flow rate measurements were carried out on 27 July 2021.

## 4. Results and Discussion

### 4.1. Methodology Outcomes

As described in the previous section, the proposed technique was applied on three river cross-sections nearby the Ceglie Messapica and Carovigno municipalities, respectively. The video sensing was carried out using a camera placed at 1.2~1.4 m in height, both perpendicularly and in parallel with respect to the flow direction (Figure 6).

The techniques explained in Section 2.1. are reported here in terms of the results of the case study. The first result is the detection of the temperature distribution using the MUSIC technique to retrieve the hyperthermia of the channel, as is undertaken with the UWB (ultra-wideband) radar when examining the reaction of human tissue and its permittivity.

**Figure 7:**
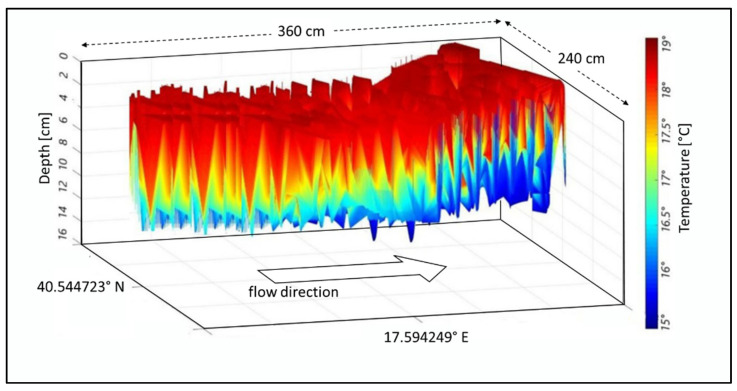
Temperature values, retrieved using the beamforming-based technique vs. depth and GPS references for cross-section A.

In our case, the riverbed is considered an organic tissue and the water flow is similar to blood flow within the arteries. Nevertheless, as the riverbed is made of reinforced concrete, the temperature tends to decrease with depth (Figure 7). These temperature values are in line with the seasonal ones. In figures where the flow direction is not explicitly indicated, it must be understood from left to right.

**Figure 8:**
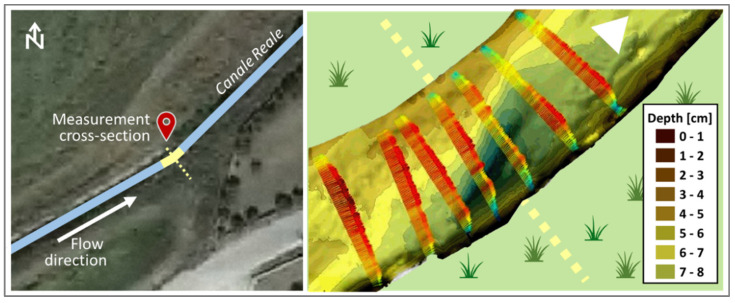
Detection of different layers and waves of the channel vs. depth for cross-section A.

The MUSIC algorithm allows separation of close values of the same quantity, such as the temperature values in Figure 7 and the wave movements (displacements) within the channel as shown in Figure 8. Such wave displacements represent the intensity of the waves along the flow direction, and as a function of the channel depth. Both these parameters, as estimated by the MUSIC techniques, can play an important role in the appraisal of river hydrodynamics.

**Figure 9:**
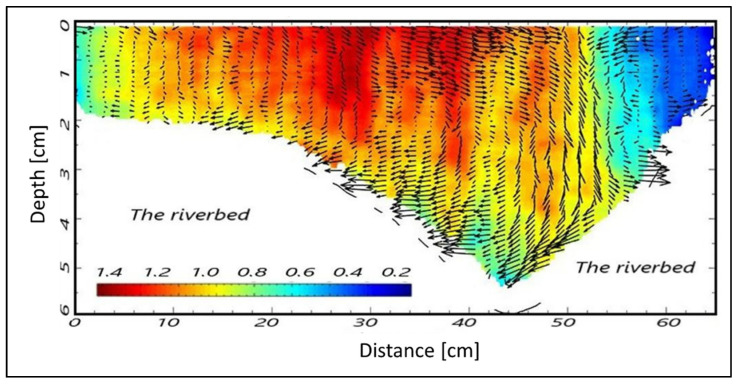
Detection of the riverbed and wave movements using Doppler processing for cross-section A.

**Figure 10:**
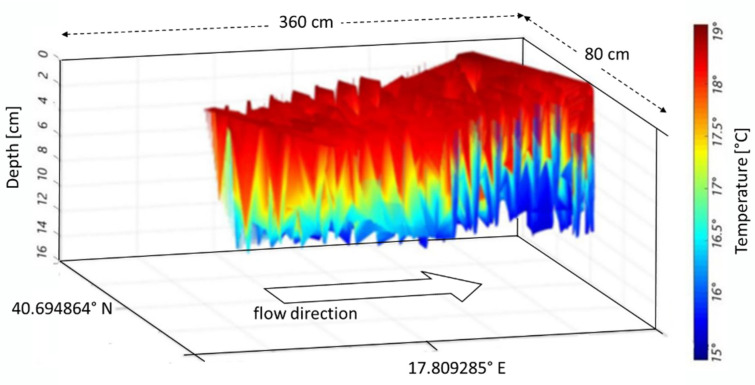
Temperature values retrieved by means of beamforming-based technique vs. depth and GPS references for cross-section B1.

Beyond MUSIC, the intention is to use the diffusion approach connected to radar sensing to extract other features, namely water waving. As reported in Section 2.1, the Doppler processing is connected to the speed/diffusion of the parameter under test. The expression of the same waves as a function of the depth is represented in Figure 9. The field of the waves within the riverbed, in terms of vectors, can be used as a first metric (indicator) to study the dynamics of rising phenomena in the channel capable of provoking extreme hydrological events such as flooding. Finally, the Doppler processing of the video imaging, allowed us to determine the water velocity as a function of the temperature (i.e., hot water and cold water) (Figure 10).

**Figure 11:**
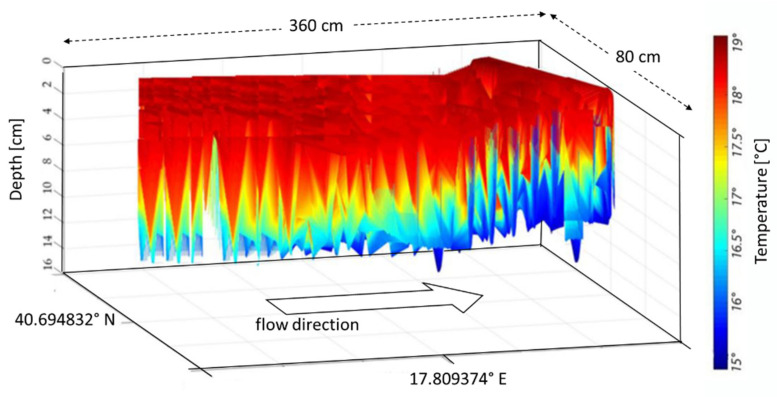
Temperature values retrieved by means of beamforming-based technique vs. depth and GPS references for cross-section B2.

The proposed methods were applied in two other sections, namely B1 and B2, to understand the efficiency and stability. Nevertheless, the plots were grouped by compared parameters in the following sections. Figure 10 and Figure 11 show the temperature in cross-sections B1 and B2. A comparison of Figure 7, Figure 10, and Figure 11 highlights a slight difference in the temperature distribution.

**Figure 12:**
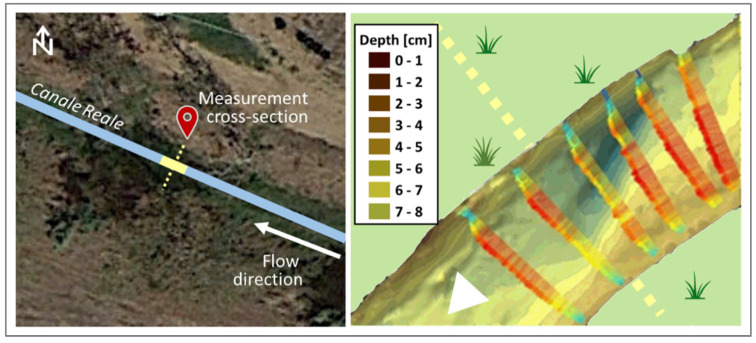
Detection of different layers and waves of the channel vs. depth for cross-section B1.

**Figure 13:**
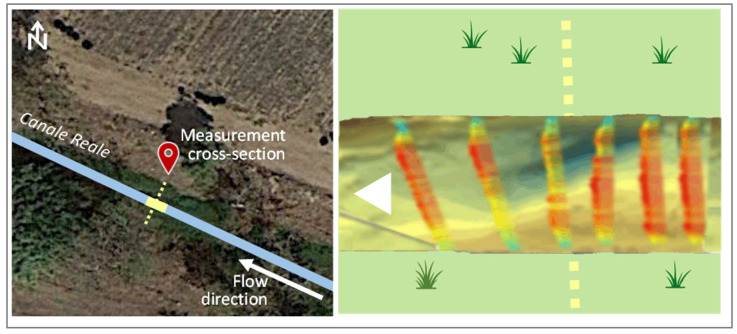
Detection of different layers and waves of the channel vs. depth for cross-section B2.

**Figure 14:**
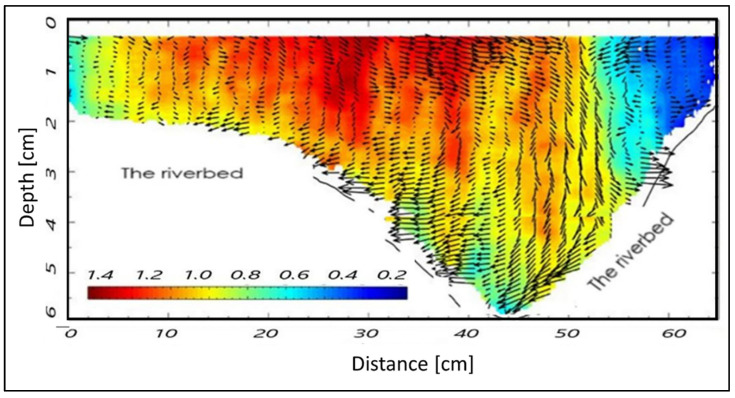
Detection of the riverbed and wave movements using Doppler processing for cross-section B1.

**Figure 15:**
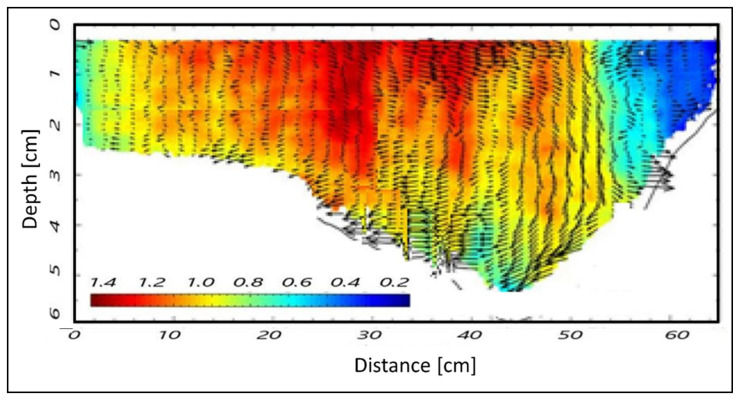
Detection of the riverbed and wave movements using Doppler processing for cross-section B2.

Even concerning the plots of layers and waves in the three cross-sections, Figure 12 and Figure 13, Figure 14 and Figure 15, and Figure 8 and Figure 9, respectively, seem to show a similar behavior of the wave movement field, even though characterized by slight differences in the estimated water depth. In particular, Figure 9 indicates a water depth of about 5.5 cm in cross-section A, while Figure 14 and Figure 15 indicate water depths of 6.0 and 5.3 cm in cross-sections B1 and B2, respectively. These few dissimilarities can be explained by different solid matter deposition and algal carpets on the channel bed.

**Figure 16:**
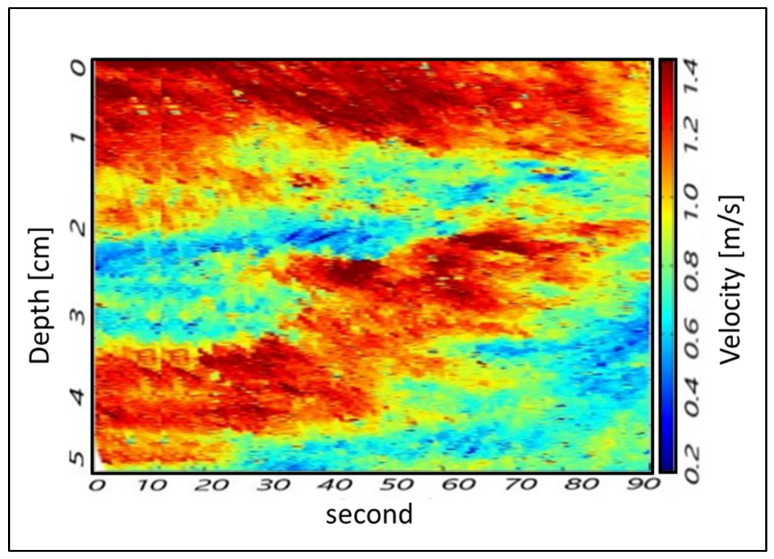
Detection of water velocity as a function of the temperature using Doppler processing: cold water—bottom; hot water—surface, for cross-section A.

Finally, the water velocities at the three cross-sections were assessed using Doppler processing, per river depth as a function of the temperature. The results show that the water flow velocities are almost the same in any of the considered cross-sections. In detail, the detected water velocities range from 0.2 to 1.4 m/s which correspond to flow rates ranging from 40 to 280 l/s and 28 to 196 l/s, in cross-sections A and B, respectively (Table 1). Figure 16 shows the water flow velocity assessed at cross-section A as an example of the methodology outcome.

As known in the literature, the more the considered variable values fall in the upper side of the range of possible values, the better the Delay and Sum beamforming (DAS), performs, providing more reliable results in terms of uncertainty. Moreover, in the case at hand, the loss of reliability of DAS is also due to unfavorable environmental conditions, such as the presence of a concrete riverbed, causing problems such as ghost or multiple sources that appear to arrive from different directions, due to successive reflections in walls.

### 4.2. Field Flow Measurements and Method Validation

In order to carry out the riverbed flow measurements using the F method, a cylinder float, 10 cm long and 3 cm in diameter, closed at its ends and filled out with little pebbles to make it travel horizontally and become partially submerged, was used. Four measurements of the float travel time were taken, and the average value was used to estimate the surface speed (Vs) representative of the investigated cross-section. By applying a quadratic corrective coefficient (F) [57], the average sectional velocity (Vm) was calculated and, finally, the transit flow rate (Q) was obtained by multiplying Vm by the average cross-section area between the ones upstream and downstream.

Measurements using the CM method were carried out with a Miniwater^®^ 20 water-flow velocity meter. Punctual velocity measurements were taken starting from the right bank of the river to the left one at a step of 10 cm and a depth of 60% of the water level, in order to approximate the segment “i” average velocity (Vmi) [58]. The total transit flow rate Q was then defined as the sum of the segment flow rates computed by multiplying each segment area (Ai) by the related Vmi.

Table 1 reports the Q values measured by the CM and F methods and the ranges of those estimated by the DAS beamforming approach. As expected, the F method overestimates the CM method by about 11% in accordance with Portoghese et al. [56]. Furthermore, by comparing the Q values obtained by the two on-site methods and the DAS beamforming, an apparent discrepancy arises. Undoubtedly, the DAS estimated Q ranges are rather wide and then a direct comparison with the related on-site measured Q is not feasible. Nevertheless, it is noteworthy that the measured values always fall within the DAS estimated ranges even though they are in the lower part. The scientific literature reports that the DAS method provides more accurate estimations in the upper part of the range of estimation. 

**Table 1:** Values of the transit flow rate (Q) measured in the cross-sections (nd = not determined).

		On-Site Direct Measures		DAS Beamforming
Cross-Section	Wet Area (m^2^)	CM Method (L/s)	F Method (L/s)	Doppler (L/s)
A	0.20	93.74	104	40 ÷ 280
B1–B2	0.14	33.90	nd	28 ÷ 196

More accurate estimations can be obtained in three ways: (i) repetition of the measurement in higher flow rates conditions, that is major velocity over 50% of the range; (ii) implementation of a weighted DAS, able to overcome the loss of reliability of DAS due to the aforementioned unfavorable environmental conditions (iii) using a delay-multiply-sum-to-standard-deviation-factor (DMSSF). This latter approach allows improvement in many types of drawbacks such as lateral resolution, signal to noise ratio (SNR), and reduces side lobes of the reconstructed image in comparison to the other conventional methods, such as DAS. The DMSSF technique will be implemented in further research.

Substantially, the on-site flow rate measurements carried out by the CM and F methods were used as true values for validating the proposed methodology. The on-site measures were repeated in three different locations along the considered river.

The layers and wave outputs from the DAS beamforming approach were considered in order to evaluate the performance of the method. This choice relies on the fact that there are interesting fluctuations in these parameters; in summary, the radar clearly detects 7 stretches (as sub-cross-sections) nearby cross-section A (Figure 8), and 6 stretches nearby cross-sections B1 and B2, respectively (Figure 12 and Figure 13). Nevertheless, these figures also show an apparent foggy background, maybe due to different solid matter deposition and algal carpets on the channel bed.

**Figure 17:**
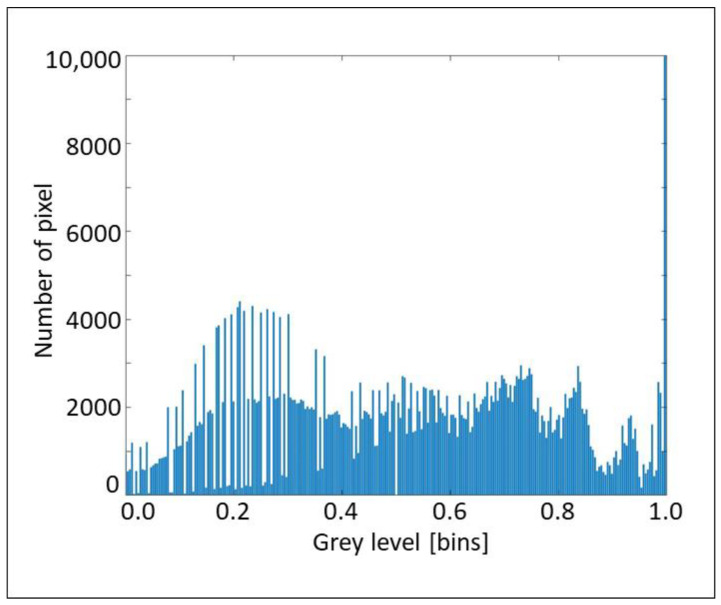
Histogram of grey scale vs. number of pixels for the section A concerning layers and waves.

**Figure 18:**
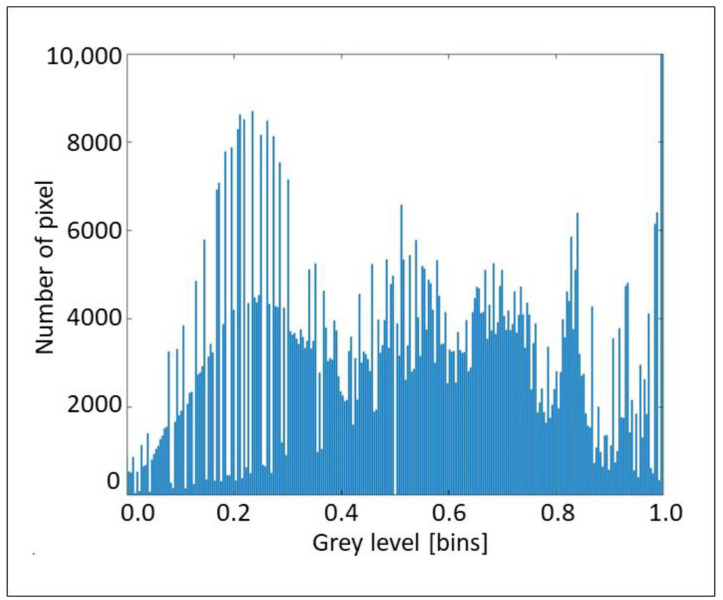
Histogram of grey scale vs. number of pixels for section B1 concerning layers and waves.

**Figure 19:**
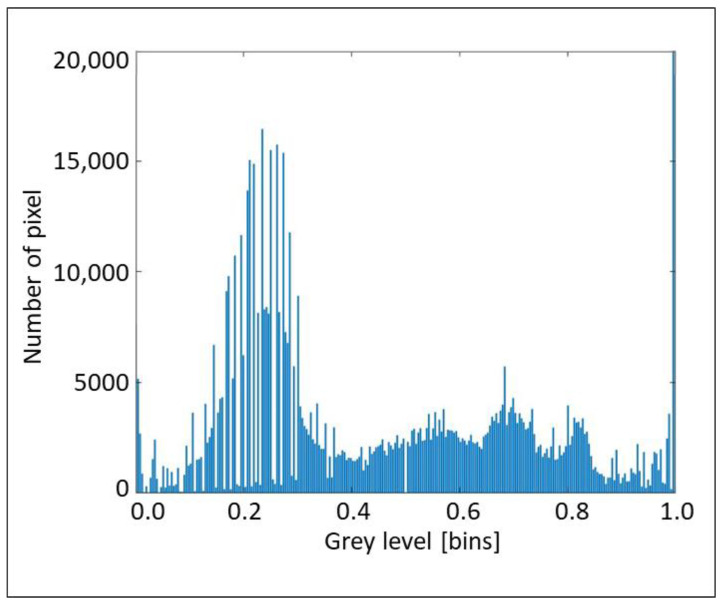
Histogram of grey scale vs. number of pixels for section B2 concerning layers and waves.

The aforementioned considerations are clearly explained by the histograms in Figure 17, Figure 18 and Figure 19, which propose the grey scale vs. the number of pixels, for the three considered cross-sections, extracted from Figure 8, Figure 12 and Figure 13, respectively. Figure 17 and Figure 18 show a great number of peaks along the abscissa, whilst Figure 19 reveals a low number of peaks due to the foggy background. Again, we can affirm the good indications from the proposed technique.

## 5. Conclusions

The growing technological improvements in video sensing systems, particularly in mobile video cameras, and the development of innovative signal processing methods and algorithms allow the use of these devices in many environmental monitoring applications.

Reliable environmental monitoring is a complex and expensive task. It often requires teams of experts moving over wide areas with unwieldy measurement devices and tools to carry out the measurements or control and calibrate on-site installed expensive devices. The chance of using cheap, small, and remotely controlled devices capable of continuously recording the dynamics of the considered environmental parameters would facilitate the monitoring tasks by reducing the related costs and providing bigger databases.

As an example, controlling the river flow using classic measurement systems, consisting of simple or automatic hydrometers located on the riverbanks, often require a person to read the current water level or acquire the recorded measurements. Nevertheless, the dynamics of the rivers often damage the device left on site or do not allow one to reach the monitoring location. More complex river flow measurement techniques, such as those using a current meter, require a significant effort both in terms of involved people and measurement gear, particularly when the riverbed size is large or the flow rate is high. Finally, these classic methods are often not applicable or fail when measuring small or null flow rates in intermittent or ephemeral river regimes.

Using small video sensors located along with the rivers’ courses at given cross-sections (e.g., bridge-mounted sensors) or placed on remotely controlled drones would enormously facilitate such a monitoring task, allowing one to carry out measurements independently from people availability, weather conditions, and time availability.

Reliable imaging processing algorithms, widely used in other technical fields, such as medical diagnostic imaging, can be suitably borrowed to deduce environmental parameter measurements from recorded videos.

In this paper, two techniques (i.e., MUSIC and Doppler processing) based on the Delay And Sum beamforming (DAS) were adopted and adapted for measuring flow rate, temperature, and wave displacement at a video sensing equipped monitoring cross-section of the Canale Reale, south Italy. In particular, the MUSIC algorithm was reliably used to provide accurate measures of temperature and water wave displacement within the considered river reach. The second technique, the Doppler processing, working as a Doppler radar, was applied to infer the diffusion of hot/cold waves in the considered river reach and extrapolate the mean water flow velocity in its middle cross-section.

The study outcomes are promising, given they were obtained by processing a short video taken by a commercial camera, although robustly post-processed. Moreover, they indicate the potentiality of the beamforming algorithm applied to hydrologic monitoring even though a methodological improvement is going to be investigated, which will be the topic of further work.

## Data Availability

Not applicable.
